# Effects of starvation on the carbohydrate metabolism in *Harmonia axyridis* (Pallas)

**DOI:** 10.1242/bio.025189

**Published:** 2017-06-12

**Authors:** Zuo-Kun Shi, Su Wang, Shi-Gui Wang, Lu Zhang, Yan-Xia Xu, Xiao-Jun Guo, Fan Zhang, Bin Tang

**Affiliations:** 1 Hangzhou Key Laboratory of Animal Adaptation and Evolution, College of Life and Environmental Sciences, Hangzhou Normal University, Hangzhou, Zhejiang 310036, China; 2 Institute of Plant and Environment Protection, Beijing Academy of Agriculture and Forestry Sciences, Beijing 100089, China

**Keywords:** Trehalase, Trehalose, Glycogen, Starvation, QRT-PCR, *Harmonia axyridis*

## Abstract

Trehalose plays an important role in energy storage, metabolism, and protection from extreme environmental conditions in insects. Trehalose is the main blood sugar in insects, and it can be rapidly used as an energy source in times of need. To elucidate the mechanisms of the starvation response, we observed the effects of starvation on trehalose and glycogen, trehalase activity, and the relative gene expression of genes in the trehalose and glycogen metabolic pathways in the invasive beetle *Harmonia axyridis*. Our results show that trehalose levels and the activities of two types of trehalases decreased significantly in the first 8 h of starvation, while the relative expression of *HaTreh1-1* increased. While trehalose remained nearly constant at a relatively high level from 8 to 24 h, glycogen levels decreased significantly from 8 h to 24 h of starvation. Likewise, glycogen phosphorylase (*HaGP*) expression was significantly higher at 12 to 24 h starvation than the first 8 h, while the expression of glycogen synthase (*HaGS*) was relatively stable. Furthermore, trehalose decreased significantly from 24 h starvation to 72 h starvation, while trehalase activities and the relative expression of some *HaTreh* genes generally increased toward the end of the starvation period. The expression of trehalose-6-phosphate synthase (*HaTPS*) increased significantly, supporting the increase in trehalose synthesis. These results show that trehalose plays a key role in the energy provided during the starvation process through the molecular and biochemical regulation of trehalose and glycogen metabolism.

## Abstract (precis)

**Summary:** Effects of starvation on the molecular and biochemical mechanisms of carbohydrate metabolism were regulated by trehalose and glycogen metabolism genes' expression changed in *Harmonia axyridis* (Pallas).

## INTRODUCTION

The beetle *Harmonia axyridis* (Pallas) (Coleoptera: Coccinellidae) is native to Asia but has been intentionally introduced to many countries as a biological control agent of pest insects (Roy et al., 2016). *Harmonia axyridis* is especially hardy, and its dramatic spread within many countries has been met with considerable trepidation. In addition to dramatically increased numbers in countries where it was introduced, *H. axyridis* has also spread to many countries where it was not intentionally released (Brown et al., 2008a). For example, not only is *H. axyridis* notoriously invasive in Europe (Adriaens et al., 2008; Brown et al., 2008a,b), it has also been reported that the global spread of *H. axyridis* has been a rapid process (Brown et al., 2011). However, this beetle is considered to be an important natural enemy of insect pests (Zappalà et al., 2013; Mirande et al., 2015), and is a focus of pest control strategies in both agriculture and forestry in Asian countries, including China (Tang et al., 2014). The hardiness of *H. axyridis* in relation to cold is an important characteristic and has been widely studied with respect to biological control applications and aspects of invasiveness (Bazzocchi et al., 2004; Berkvens et al., 2010; Pervez and Omkar, 2006; van Lenteren et al., 2008).

Trehalose is a non-reducing sugar that consists of two glycosidically linked glucose units, and although it is found in a diverse array of taxa (e.g. bacteria, yeast, fungi, nematodes, plants, insects, and other invertebrates), it is absent in mammals (Elbein, 1974; Elbein et al., 2003; Frison et al., 2007; Wingler, 2002). Glycogen is a polysaccharide that is widely distributed in microorganisms and animal cells, where its main role is as a reserve carbohydrate (Tang et al., 2012a,b). Carbohydrate storage is required in most cell types so that they can be degraded for energy production when necessary. Previous studies involving trehalose and glycogen metabolism have shown that the synthesis of trehalose from glycogen or from glucose in insects involves several enzymes (Elbein et al., 2003; Kunieda et al., 2006; Montooth et al., 2003; Tang et al., 2010), where glucose-6-phosphate (G6P), glucose-1-phosphate (G1P), and uridine diphosphate (UDP)-glucose are the integral substrates or byproducts in insect energy metabolism. Some of enzymes (or genes) involved in insect energy metabolism are trehalase (Treh or TRE), hexokinase (HK), glucose-6-phosphatase (G-6-pase), glucophosphatase (PGM), glycogen phosphorylase (GP), glycogen synthase (GS), UDP-glucose pyrophosphorylase (UGPase), trehalose-6-phosphate synthase (TPS), and trehalose-6-phosphate phosphatase (TPP) (Tang et al., 2012a; Yang et al., 2017). Trehalose and glycogen are the central sources of glucose and glucose-6-phosphate in insects and may be key intermediate products (Tang et al., 2012a). Glycogen can be degraded by GP, PGM, and UGPase, into glucose-6-P and UDP-glucose, thereby entering into the trehalose metabolic pathway.

Energy metabolism in insects is similar to energy metabolism in other animals; however, the synthesis and utilization of trehalose is unique to insect energy metabolism in that the blood sugar in insects is trehalose instead of glucose (Friedman, 1978; Silva et al., 2004; Terra and Ferreira, 1994; Thompson, 2003; Tang et al., 2010). Trehalose and glycogen are the key energy sources in insects and are known to play an important role in physiological adaptation (Qin et al., 2012; Tang et al., 2012b). Glycogen accumulates during the diauxic phase of growth or in response to carbon, nitrogen, sulfur, or phosphorus limitations and is hydrolyzed under conditions of carbon starvation (Johnston and Carlson, 1992). Additionally, not only is trehalose one of the primary energy stores in insects vital for both movement and development, it can also play a protective role, protecting proteins and cellular membranes from a variety of environmental stresses (e.g. desiccation, dehydration, heat, freezing, and oxidation) (Bale and Hayward, 2010; Elbein et al., 2003; Friedman, 1978; Khani et al., 2007; Thompson, 2003), and has been shown to be used rapidly in the face of stressful conditions (Thompson, 2003; Tang et al., 2014).

For survival, as it is vital that animals maintain a positive balance between the energy gained from feeding and energy allocated to activity and growth, as energy stores are ubiquitous to all living organisms. The fat body and hemolymph are important for insects energy storage (Schilman and Roces, 2008), where trehalose has been shown to be the main freely circulating sugar in the hemolymph of insects (Thompson, 2003). In accordance with previous knowledge, trehalose levels in the hemolymph of insects are expected to depend on both the quantity of carbohydrates ingested and the quantity metabolized to cover the energy needs of a given individual (Schilman and Roces, 2008).

Insects, often under threat of starvation, have a greater capacity to survive starvation by undergoing a suite of physiological changes. In insects, starvation during the larval stage has been shown to reduce adult metabolic rates (Wang et al., 2016a) resulting in an increase in the energy stores of glycogen, triglycerides (Wang et al., 2016a), glucose, and trehalose in adults (Kim and Hong, 2015; Wang et al., 2016a; Zauner et al., 2000) during the intial stage of starvation; but it can result in a decrease in the content of trehalose and glucose with increasing time of starvation (Laparie et al., 2012; Schilman and Roces, 2008). It is well known that starvation has been shown to be regulated by insulin and 20-hydroxyecdysone (20E) (Keshan et al., 2016; Kim and Hong, 2015).

*Harmonia axyridis* is able to survive in a variety of environmental conditions and has a worldwide distribution. Predatory insects such as *H. axyridis* can survive and can even thrive under conditions of starvation, when there are few insect pests to prey upon (Shi et al., 2016; Tang et al., 2014). In a previous study (Tang et al., 2014), we found that sprint speed and maximum moving distance increased and pause frequency decreased in adult *H. axyridis* starved for 8 h compared with control (0 h) adults. In contrast, the mRNA expression of trehalase genes, including *HaTreh1-1* and *HaTreh1-2*, increased quickly in adults starved from 8 to 18 h, particularly with *HaTreh1-1*, which was expressed 289-fold in adults starved for 18 h in comparison to control adults. In contrast, the expression of trehalose-6-phosphate synthase (*HaTPS*) decreased prior to 12 h and then increased (Tang et al., 2014). To date, more than seven trehalase genes have been cloned (Shi et al., 2016; Tang et al., 2014), as well as one *TPS* gene (Qin et al., 2012), one *GP* gene, and one *GS* gene. We want to know how these genes regulated the balance of trehalose and glycogen under starvation condition. Increasing our knowledge of the genes involved in the insect response to starvation will help to elucidate the function of trehalose metabolism in starvation.

## RESULTS

### Effects of starvation on trehalose and glycogen contents

**Fig. 1.**
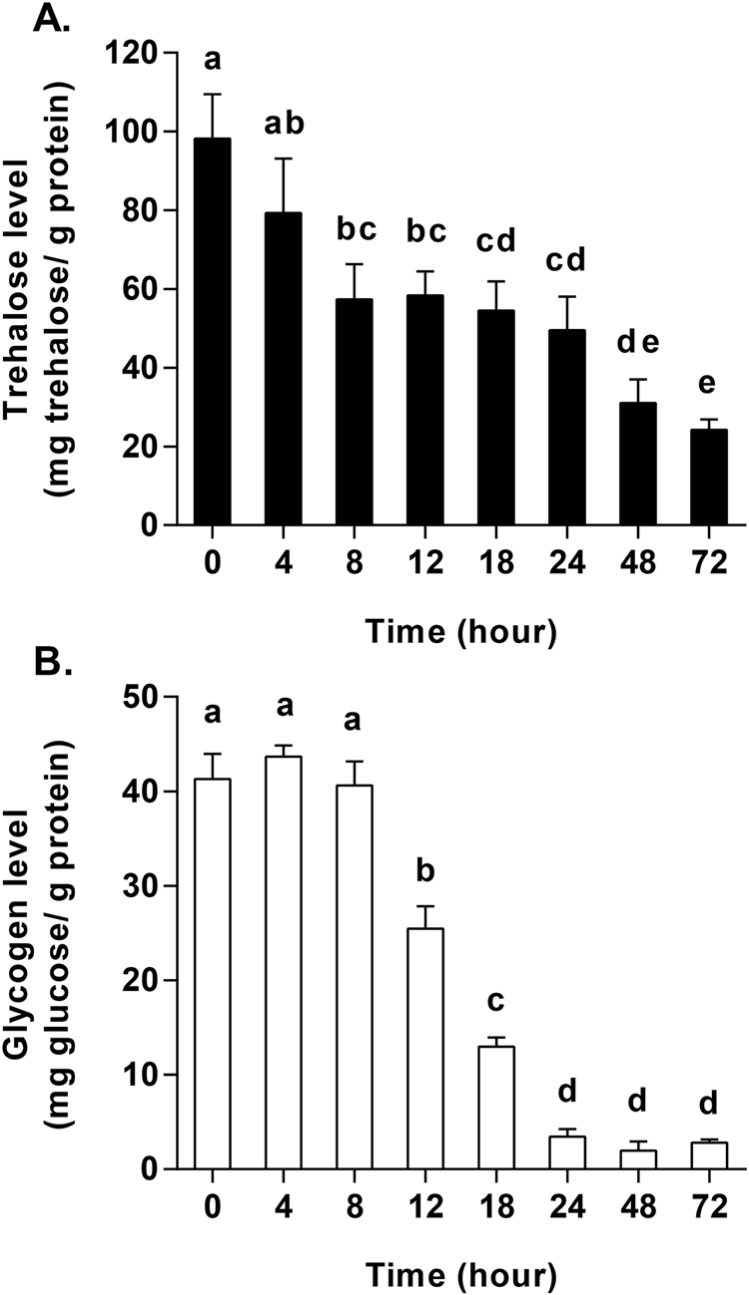
**Changes in trehalose and glycogen in adult non-melanic *Harmonia axyridis* in response to starvation (0–72 h).** (A) Changes in trehalose during starvation. (B) Changes in glycogen during starvation. Bars with different letters indicate significant differences (*P*<0.05; one-way ANOVA test). Data are presented as means±s.d. (*n*=3).

Here, we compared trehalose and glycogen contents in response to different starvation periods in the beetle *H. axyridis*. In general, our results show that both trehalose and glycogen contents decreased as starvation time increased. The results showed that the highest level of trehalose content observed [98.21±11.31 mg trehalose/g protein (mean±s.d.)] was at 0 h starvation (*F*_7, 16_=22.74, *P*<0.001) (Fig. 1A), while the highest level of glycogen content observed (43.66±1.18 mg glucose/g protein) was at 4 h starvation (*F*_7, 16_=352.93, *P*<0.001) (Fig. 1B). Trehalose content decreased significantly from 0 h to 8 h starvation, leveled off at a relatively high quantity from 8 h to 24 h (>49.49 mg trehalose/g protein), and the continued to decrease for the duration of the experiment to 24.25±2.65 mg trehalose/g protein at 72 h starvation (Fig. 1A). In addition, the results show that the content of glycogen decreased more than 40 μg glucose/mg protein during the first 8 h, and then decreased significantly to the lowest level observed (1.97±0.98 mg glucose/g protein) at 48 h starvation (Fig. 1B).

### Effects of starvation on soluble and membrane-bound trehalase activity

**Fig. 2.**
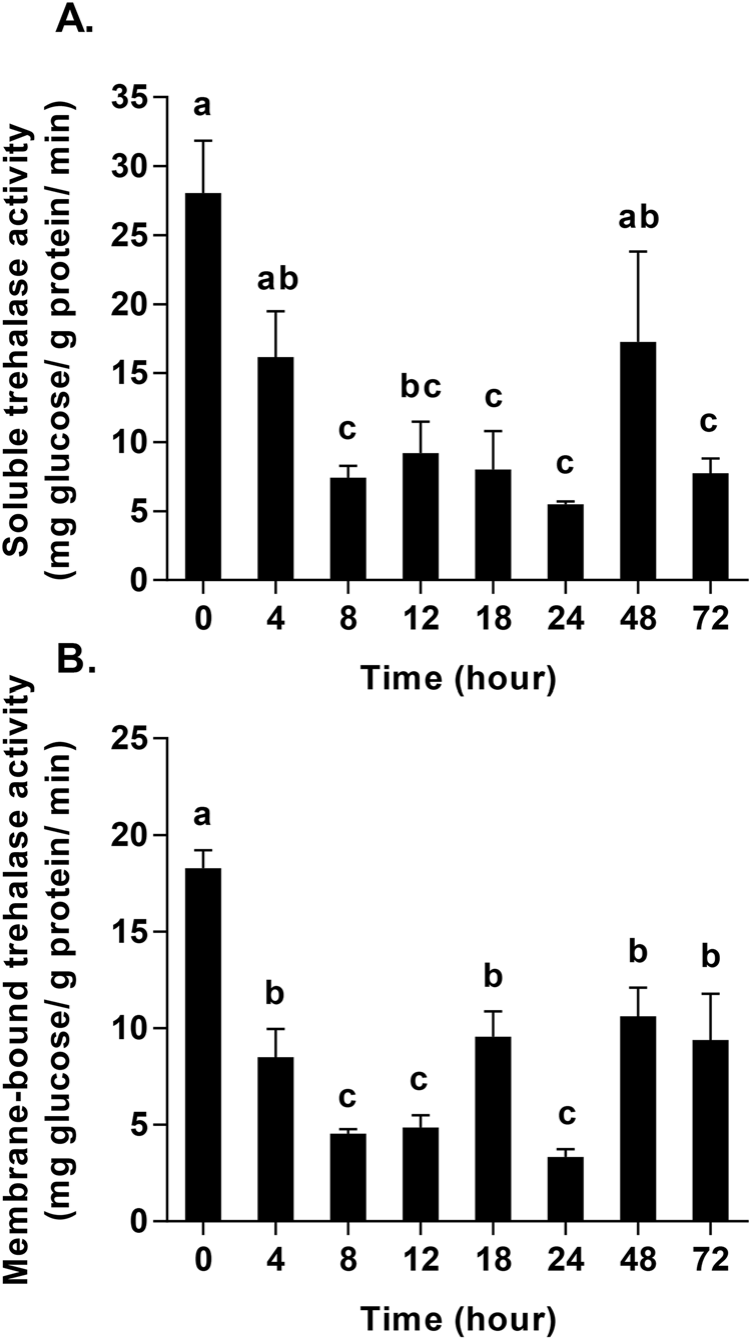
**Changes in trehalase activity in *Harmonia axyridis* in response to starvation (0–72 h).** (A) Changes in soluble trehalase activity during starvation. (B) Changes in membrane-bound trehalase activity during starvation. Bars with different letters indicate significant differences (*P*<0.05; one-way ANOVA test). Data are presented as mean±s.d. (*n*=3).

Here, we assessed effects of starvation on the activities of soluble and membrane-bound trehalase and found that the two types of trehalase displayed a similar trend during the starvation process. Generally, trehalase activity was at its highest level at the beginning of starvation and decreased from 0 h to 8 h. The results show that soluble trehalase decreased significantly from 28.06±3.79 mg trehalose/g protein/min at 0 h to 7.41±0.889 mg trehalose/g protein/min at 8 h, and leveled off at approximately 5.51±0.21 mg trehalose/g protein/min from 8 h to 24 h, followed by a significant increase to 17.26±6.54 mg trehalose/g protein/min at 48 h starvation and 17.76±1.05 mg trehalose/g protein/min at 72 h starvation (*F*_7, 16_=15.92, *P*<0.001) (Fig. 2A). Additionally, the activity of membrane-bound trehalase also decreased significantly at each time point, from 18.28±0.93 mg trehalose/g protein/min at 0 h to 4.55±0.22 mg trehalose/g protein/min at 8 h, after which activity increased significantly to more than 9.38±2.39 mg trehalose/g protein/min at 18 h, 48 h, and 72 h starvation (*F*_7, 16_=40.88, *P*<0.001) (Fig. 2B).

### Changes in mRNA expression of soluble trehalases and membrane-bound trehalases during starvation

**Fig. 3.**
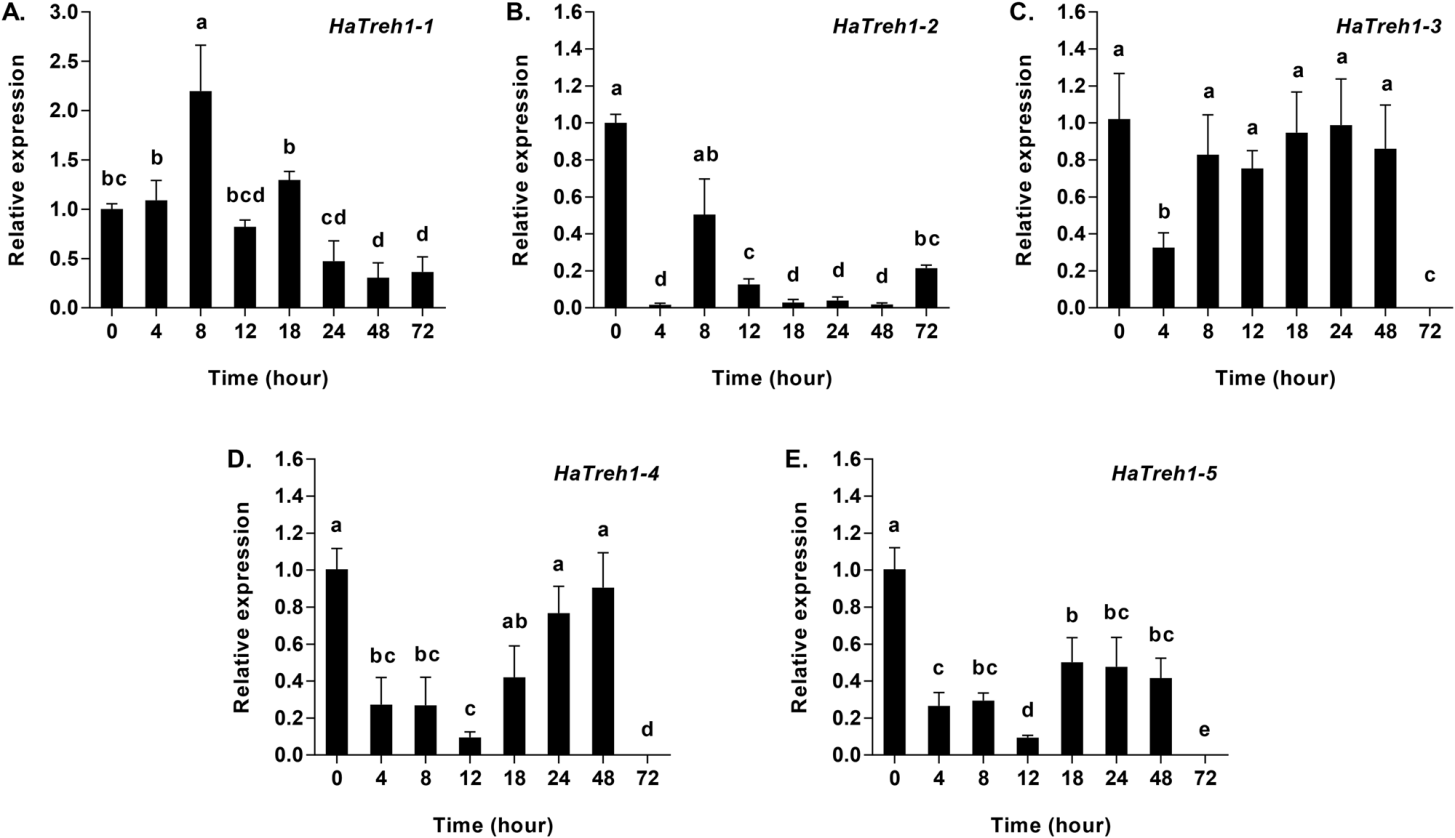
**Quantitative mRNA expression of five soluble trehalase genes mRNA in *Harmonia axyridis* in response to starvation (0–72 h).** (A) mRNA expression of *HaTreh1-1*. (B) mRNA expression of *HaTreh1-2*. (C) mRNA expression of *HaTreh1-3*. (D) mRNA expression of *HaTreh1-4*. (E) mRNA expression of *HaTreh1-5*. Gene expression is relative to the expression of the endogenous control Harp49 (*H. axyridis* ribosomal protein 49 gene), measured via qRT-PCR. Data are presented as mean±s.d. (*n*=3). Bars with different letters indicate significant differences (*P*<0.05; one-way ANOVA test).

**Fig. 4.**
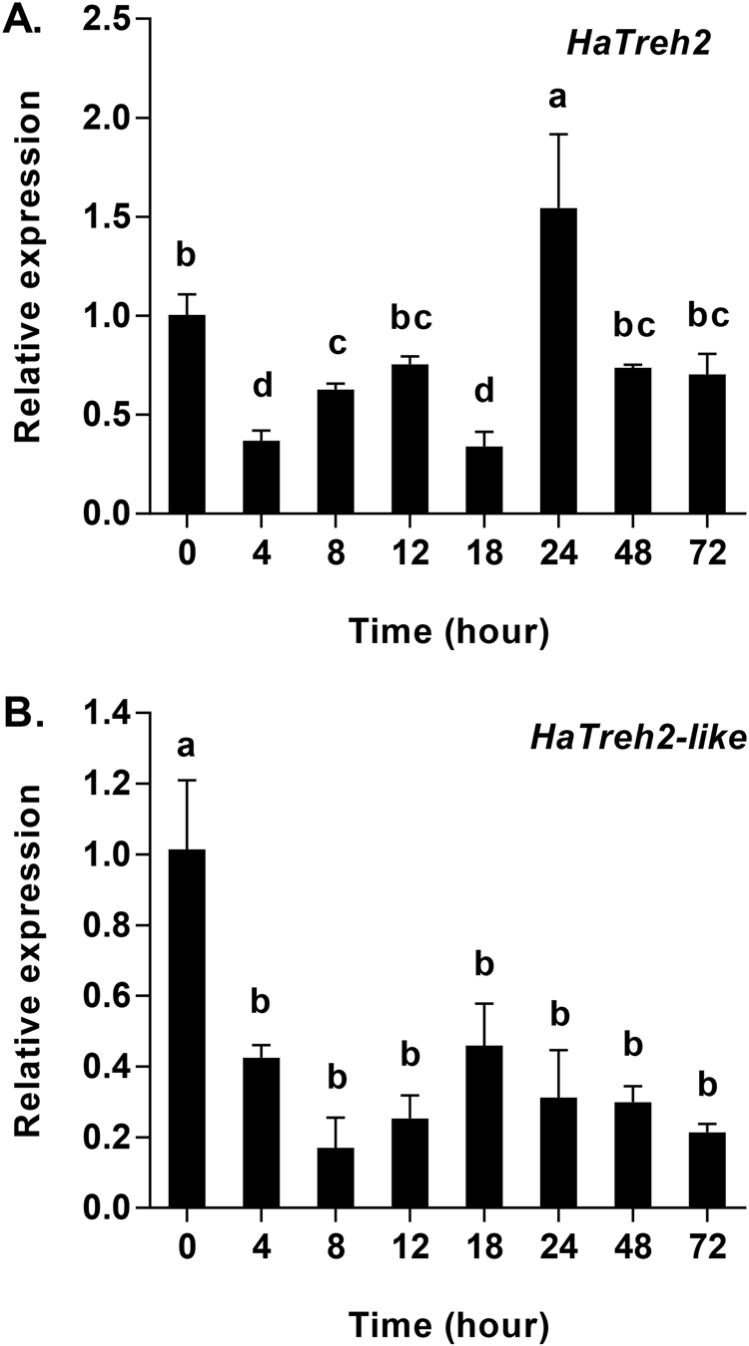
**Quantitative mRNA expression of two membrane-bound trehalase genes in *Harmonia axyridis* in response to starvation (0–72 h).** (A) mRNA expression of *HaTreh2*. (B) mRNA expression of *HaTreh2-like*. Gene expression is relative to the expression of the endogenous control Harp49 (*H. axyridis* ribosomal protein 49 gene), measured via qRT-PCR. Data are presented as mean±s.d. (*n*=3). Bars with different letters indicate significant differences (*P*<0.05; one-way ANOVA test).

Similar to the enzymatic activities of both the soluble and membrane-bound trehalases, the mRNA expression of most soluble and membrane-bound trehalase genes also decreased during the eight starvation, with the exception of *Treh1-1* at 8 h and 18 h (*F*_7,16_=25.82, *P*<0.001), and *Treh2* at 24 h (*F*_7, 16_=35.72, *P*<0.001) (Figs 3A and 4B). The expression of *Treh1-2* was lowest at 0 h (control group) and displayed relatively high levels only 8 h and 72 h starvation (*F*_7, 16_=43.14, *P*<0.001) (Fig. 3B). The expression of *Treh1-3* decreased significantly from 0 h to 4 h, followed by increased expression for the duration of the experiment (*F*_7, 16_=351.48, *P*<0.001) (Fig. 3C). The mRNA expression of *Treh1-4* (*F*_7, 16_=244.26, *P*<0.001) and *Treh1-5* (*F*_7, 16_=232.82, *P*<0.001) displayed similar downward trends during the first 12 h of the experiment, whereupon the expression of *Treh1-4* increased at each time point from 18 h to 48 h and the expression of *Treh1-5* maintained relatively high levels from 18 h to 48 h. The levels of *Treh1-4* and *Treh1-5* mRNA were observed to be at their lowest at 72 h starvation (Fig. 3D,E).

The results showed that trends in the expression of *Treh2-like* and *Treh2* mRNA were different. The expression of *Treh2-like* mRNA in starved *H. axyridis* adults was lower than in the control group, and was lowest in adults starved for 8 h, while not differing significantly between the 4 h to 72 h time points (*F*_7, 16_=20.23, *P*<0.001) (Fig. 4B). By contrast, the expression of *Treh2-like* mRNA differed according to the length of the starvation period. The expression of *Treh2* mRNA decreased significantly from 0 h to 4 h, increased at 8 h and 12 h, was the lowest at 18 h, increased to the highest level of expression at 24 h, decreased significantly from 24 h to 72 h, and finally maintained relatively high levels at 48 h and 72 h, which were not significantly different from that at 0 h (Fig. 4A).

### Effects of starvation on TPS expression

**Fig. 5.**
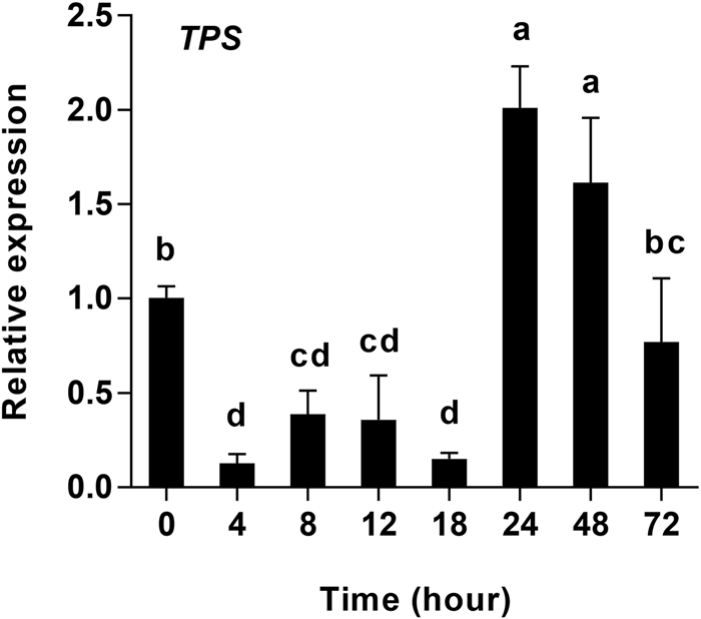
**Quantitative mRNA expression of trehalose-6-phosphate synthase (*HaTPS*) in *Harmonia axyridis* in response to starvation (0–72 h).** Gene expression is relative to the expression of the endogenous control Harp49 (*H. axyridis* ribosomal protein 49 gene), measured via qRT-PCR. Data are presented as mean±s.d. (*n*=3). Bars with different letters indicate significant differences (*P*<0.05; one-way ANOVA test).

The mRNA expression of the TPS gene identified in *H. axyridis* was calculated for all eight starvation periods. The expression of TPS mRNA was different over the course of the starvation experiment (*F*_7, 16_=32.75, *P*<0.001). The results showed that TPS expression between 4 h to 18 h was lower than at 0 h of starvation, and did not differ significantly between the four time points in this period. TPS expression increased significantly from 18 h to 24 h, peaked at 24 h, and decreased from 24 h to 72 h, maintaining a higher level at 48 h, and had significant difference with control group (0 h) (Fig. 5).

### Effects of starvation on *HaGP* and *HaGS* expression

**Fig. 6.**
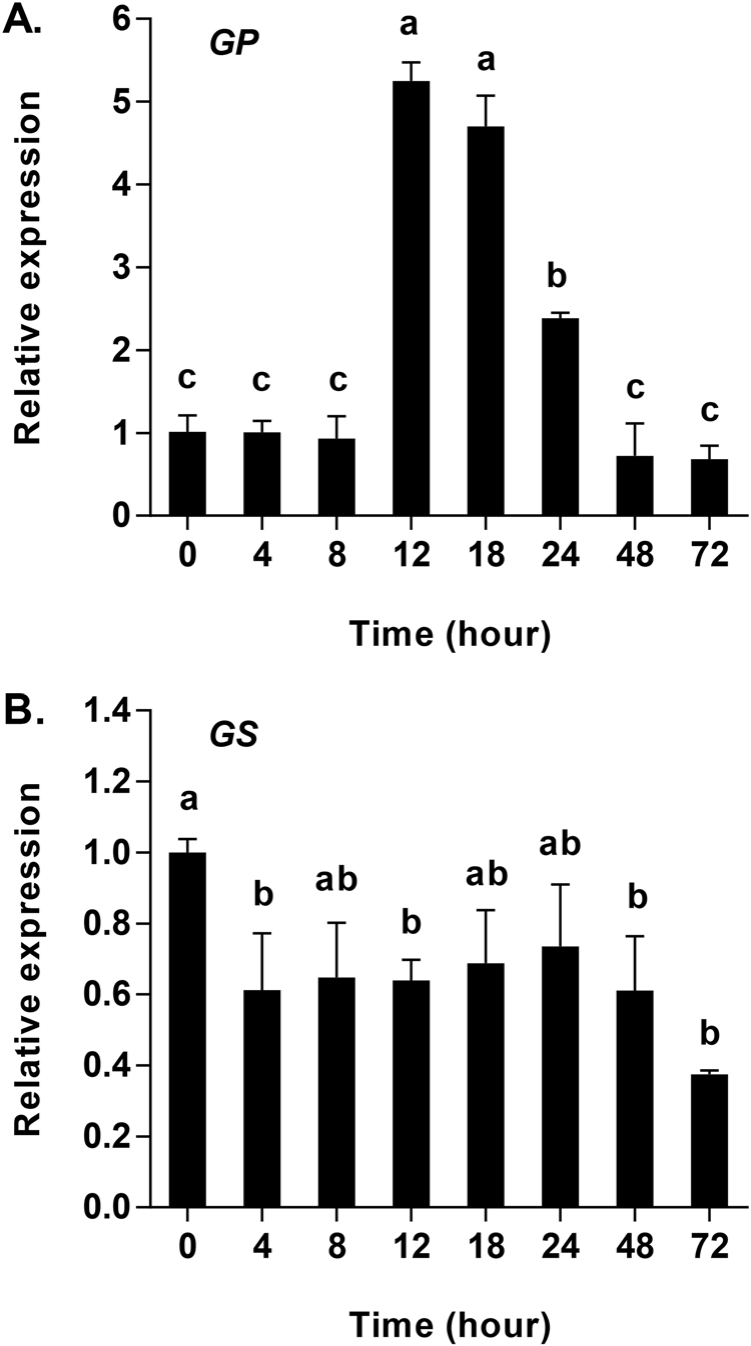
**Quantitative mRNA expression of glycogen phosphorylase (GP) and glycogen synthase (GS) in *Harmonia axyridis* in response to starvation (0–72 h).** (A) mRNA expression of *HaGP*. (B) mRNA expression of *HaGS*. Gene expression is relative to the expression of the endogenous control Harp49 (*H. axyridis* ribosomal protein 49 gene), measured via qRT-PCR. Data are presented as mean±s.d. (*n*=3). Bars with different letters indicate significant differences (*P*<0.05; one-way ANOVA test).

The mRNA expression of glycogen phosphorylase (*HaGP*) and glycogen synthase (*HaGS*) were detected via qRT-PCR (Suann et al., 2015). As a result, *HaGP* increased while *HaGS* expression decreased during the starvation period (Fig. 6). Changes in the mRNA expression of *HaGP* were not significantly different at 0 h, 4 h, 8 h, 48 h and 72 h, and were fivefold higher than in the control group at 12 h and 18 h. *HaGP* expression also decreased significantly from 12 h to 48 h, reaching the lowest level of expression (*F*_7, 16_=164.85, *P*<0.001) (Fig. 6A). The expression of *HaGS* mRNA was lower than the control group from 4 h to 72 h of starvation, and was significantly lower at 0 h of starvation than at 4 h and 72 h of starvation (*F*_7, 16_=5.48, *P*=0.002) (Fig. 6B). In addition, there were no significant differences from 4 h to 72 h of starvation.

## DISCUSSION

Trehalases can catalyze the hydrolysis of one mole of trehalose to two moles of glucose and are used for the uptake or utilization of trehalose in the hemolymph. In insects, there are two different types of trehalases: a soluble trehalase (Treh1) and a membrane-bound trehalase (Treh2), which includes more than one potential transmembrane helix (Chen et al., 2010; Tang et al., 2012a). In our study, the concentrations of trehalose and glycogen decreased with increasing starvation time (Fig. 1), similar to the results of a previous study showing that low deprivation and high deprivation (i.e. immobility and active behavior) in ants resulted in a significant decrease in trehalose in response to 11–13 days of carbohydrate deprivation (Schilman and Roces, 2008). When exposed to high sugar deprivation, the levels of trehalose and fructose in immobile ants were higher in comparison to active ants, while there was no significant difference in glucose levels. These results showed that insect movement was fueled by energy from trehalose and fructose (Schilman and Roces, 2008). Likewise, the beetle *H. axyridis* was starved under normal conditions, and as they needed to find food to mitigate starvation, the sprint speed and maximum moving distance were observed to increase, while pause frequency decreased when beetles were starved for 8 h in comparison to control (0 h) adults (Tang et al., 2014). Under such conditions, and as the need for energy increases, we found that this energy comes from trehalose prior to 8 h of starvation in adult *H. axyridis* (Fig. 1A), while trehalose levels off and glycogen levels decreased significantly (ninefold lower) from 8 h to 24 h (Fig. 1). These results indicate that glycogen was the main energy resource consumed from 8 h to 24 h of starvation.

Glycogen can be utilized as a second form of energy storage during short periods of starvation, as trehalose continued to decrease from 48 h to 72 h starvation while glycogen kept at a lower level (Fig. 1). In another study, levels of trehalose increased after 12 h to 48 h of starvation, while the expression of insulin-like peptides (ILPs) decreased in *Spodoptera exigua* (Hübner) (Lepidoptera: Noctuidae) larvae (Kim and Hong, 2015). Furthermore, trehalose levels increased significantly when the expression of ILP genes was knocked downed via RNAi in both *S. exigua* and *Drosophila melanogaster* (Kim and Hong, 2015; Zhang et al., 2009). On the other hand, the concentration of trehalose in fatty acid titers and hemolymph declined gradually over six days of starvation in the moth *Heliothis virescens* (Fabricius) (Lepidoptera, Noctuidae) (Foster and Johnson, 2010). Other previously published studies have also demonstrated that glycogen and trehalose play an important role in starvation and conditions of food deprivation by regulating the expression of the insulin receptor (InR) via insulin and 20E (Keshan et al., 2016; Matsuda et al., 2015). There have also been shown to be differences in the effects of starvation on trehalose and glycogen according to development, where trehalose and glycogen decreased in adults (in comparison to larvae) in response to starvation, as in the present study (Fig. 1) (Keshan et al., 2016; Wang et al., 2016a,b).

In a previously published starvation experiment of *H. axyridis* adults, the expression of *HaTPS* decreased during the first 12 h and continued to increase (Tang et al., 2014), while in the present study, *HaTPS* expression did not significantly change from 4 h to 18 h of starvation (Fig. 5). Moreover, *HaTPS* expression increased significantly from 24 h to 48 h in comparison to 0 h, and reached a relatively high level at 72 h of starvation (Fig. 5). These results indicate that trehalose may be synthesized primarily between 24 h to 72 h in the face of starvation, and that other sugars play the role of opposing starvation and transferred to trehalose. Elucidating the other potential storage sugars involved in the starvation response of *H. axyridis* will require further research.

It has been suggested that the most important function of trehalase is to facilitate the uptake and utilization of trehalose from the blood (Azuma and Yamashita, 1985a,b; Tang et al., 2008; Zhao et al., 2016). The results of a previous study showed that the sprint speed after 4 h of hunger was lower than at 8 h (Tang et al., 2014), indicating an initial difficulty in adapting to starvation in adults of *H. axyridis*. As the duration of starvation increased, sprint speed was shown to decrease further and further, indicating that available energy was also decreasing (Tang et al., 2014). This finding is in agreement with our results of the levels of *HaTPS* and *HaTreh1-1* expression, as well as the activities of soluble and membrane-bound trehalases observed. Our observation that the expression of *HaGP* increased in response to starvation at 12 h (Fig. 6A) was in agreement with the observations of changes in trehalose and glycogen contents (Fig. 1) as well as the expression of *HaGP* and *HaGS*. In another study, the mRNA expression of glycogen synthase decreased and glycogen phosphorylase increased in response to short-term time starvation (i.e. 6 h, 12 h, and 24 h) in *S. exigua* (Tang et al., 2012a,b). In addition, seven trehalase genes in *H. axyridis* comprised the total soluble trehalase and membrane-bound trehalase activities, where each trehalase gene plays a distinctive role over the course of the starvation period. For example, *HaTreh1-1* played a role in degrading trehalose during the first 8 h of starvation (Fig. 3A), while *HaTreh1-3*, *HaTreh1-4*, *HaTreh1-5*, and *HaTreh2* appear to govern changes in levels of trehalose at 24 h and 48 h of starvation (Figs 3 and 4A). In summary, trehalase, TPS, GP, and GS work together to regulate changes in trehalose and glycogen levels in response to starvation stress.

In this study, adults of *H. axyridis* were put into a plastic culture dish at the beginning of the starvation experiment, where adults are able to move but not fly. In locusts, it has been previously reported that trehalose levels changed immediately when insects began to fly (Van der Horst et al., 1978). The ability of *H. axyridis*, which has a worldwide distribution, to survive in the face of adverse conditions is very strong. As it is considered to be an invasive species in some countries, studies of the physiological and biochemical underpinnings of trehalose regulation in the face of starvation could be useful to assist in the control of invasions.

## MATERIALS AND METHODS

### Experimental insects

*Harmonia axyridis* were collected from Wangjiayuan village, Beijing, China. The experimental population had been maintained in our laboratory for more than 2 years, fed with *Aphis medicaginis* (Koch) and maintained at 25±1°C. Melanic and non-melanic populations were each set up and maintained under the following conditions: 25°C, 70% relative humidity, and a 16 h light:8 h dark photoperiod. The developmental stages were synchronized at each molt by collecting new larvae, pupae, or adults by feeding with fresh *A. medicaginis* daily. Abdominal tissues from the different developmental stages were dissected in insect saline containing 0.75% NaCl and stored at −80°C until further analysis. For all starvation treatments, 7-day-old adults from the non-melanic group were used.

### Starvation treatments

Eight treatments (i.e. starvation periods) were used to observe the effects of starvation over time: 0 h (control), 4 h, 8 h, 12 h, 18 h, 24 h, 48 h, and 72 h. More than ten adults were added to a plastic culture dish (Φ9 cm) for each starvation treatment. All starvation experiments were repeated three to five times for each treatment.

### Preparation of carbohydrate extracts

Five to seven adult beetle individuals (with elytra removed) were placed in a 1.5 ml centrifuge tube. After adding 200 μl of 20 mM phosphate buffered saline (PBS, pH 6.0), tissues were homogenized at 0°C (TGrinder OSE-Y20 homogenizer; Tiangen Biotech Co., Ltd., Beijing), followed by sonication for 30 s (VCX 130PB, Sonics, Newtown, CT, USA). PBS was added (800 μl) and homogenates were centrifuged at 12,000 ×***g*** at 4°C for 10 min. Precipitates were removed and aliquots of the supernatant were assayed to determine protein concentration using the protein-dye binding method (Bio-Rad, Hercules, CA, USA) with bovine serum albumin as a standard. Then, 500 μl of supernatant was added to a 1.5 ml tube and subsequently boiled, after which the solution was centrifuged at 12,000 ×***g*** for 10 min to remove any residual protein. The supernatant was equally split between two tubes: one was directly subjected to a glycogen content assay, and the other was processed for the measurement of trehalose. Because glycogen is unstable in a strong acid but stable in a strong alkaline condition, and glucose is unstable under a strong acid or alkaline environment, while trehalose is very stable in acid or alkaline condition, in order to measure trehalose, we first hydrolyzed glycogen into glucose in sulfuric acid (H_2_SO_4_) under heating conditions. Subsequently, total glucose was decomposed the under alkaline conditions. Thus, the resulting supernatant contained trehalose without other contaminating carbohydrates and proteins. The details of trehalose and glycogen assays are as follows.

### Measurement of trehalose and glycogen contents

Trehalose content was estimated using a modified version of a previously described protocol (Ge et al., 2011). Briefly, 50 μl of supernatant was added to a 1.5 ml tube, 50 μl 1% H_2_SO_4_ was added, and the tube was incubated at 90°C in a water bath for 10 min to hydrolyze glycogen, after which it was cooled on ice for 3 min. Subsequently, 50 μl of 30% potassium hydroxide was added to decomposed glucose, and the supernatant was again incubated in water at 90°C for 10 min and cooled on ice for 3 min. Next, 4 volumes of 0.2% (M/V) anthrone (Sigma, Shanghai, China) in 80% H_2_SO_4_ solution were added after it was cooled on ice for 3 min and the supernatant boiled for 10 min. After cooling, 200 μl of the reaction solution was placed into a 96-well plate and the absorbance at 620 nm was determined using a SpectraMax M5 (Molecular Devices, Sunnyvale, CA, USA). Trehalose content was calculated based on a standard curve and was expressed as mg trehalose per g total protein.

Glycogen content was measured as described by Santos et al. (2008). Briefly, 100 μl supernatant (from section 2.3) was incubated for 4 h at 37°C in the presence of 20 μl (1 U) amyloglucosidase (EC 3.2.1.3; Sigma-Aldrich) diluted in 100 mM sodium acetate (pH 5.5) to hydrolyze glycogen. The amount of glucose generated from glycogen was determined using a Glucose Assay Kit (GAGO20-1KT; Sigma-Aldrich) following the manufacturer's instructions. Controls were prepared in the absence of enzyme, and the quantity of glycogen was calculated by excluding endogenous glucose. Glycogen content was calculated as mg glucose per g total protein.

### Soluble trehalase and membrane-bound trehalase activity assay

Trehalase activity was determined as a previously described (Tatun et al., 2008). Briefly, three abdominal tissues adult beetles were combined with 200 μl 20 mM PBS (pH 6.0) in a 1.5 ml tube and homogenized at 0°C (TGrinder OSE-Y20 homogenizer, TIANGEN, China), and subsequently sonicated for 30 s (VCX 130PB; Sonics). PBS was added (800 μl) to the homogenate, the solution was centrifuged at 1000 ×***g*** at 4°C for 10 min, cuticle debris was removed, and the resulting supernatant was centrifuged again at 105,000 ×***g*** and 4°C for 60 min (CP100MX; Hitachi, Tokyo, Japan). The supernatant fraction was transferred to a new tube and directly used in the measurement of soluble trehalase activity. The precipitate fraction was washed twice with PBS and subsequently suspended in 200 μl PBS to measure membrane-bound trehalase. The protein concentration in each sample was determined prior to the trehalase assay using a protein-dye binding method (Bio-Rad) with bovine serum albumin as a standard. For the trehalase activity assay, the reaction mixture (250 μl) consisted of 62.5 μl of 40 mM trehalose (Sigma, USA) in 20 mM PBS (pH 6.0), 50 μl of either the soluble or membrane-bound trehalase fraction, and 137.5 μl PBS. The mixture was incubated at 37°C for 30 min, and the reaction was stopped by heating in boiling water for 5 min. Coagulated protein was removed by centrifugation at 12,000 ×***g*** at 4°C for 10 min. An aliquot of the resulting supernatant was used to measure the amount of glucose using a Glucose Assay Kit (GAGO20-1KT, Sigma-Aldrich) following the manufacturer's instructions; data were expressed as mg of glucose per g protein per min.

### RNA extraction, cDNA synthesis, and quantitative real-time PCR

Total RNA was extracted from abdominal tissues using Trizol (Invitrogen, Waltham, MA, USA). First-strand cDNA synthesis was carried out using a PrimeScript^®^ RT reagent Kit with gDNA Eraser (TaKaRa, Shiga, Japan) according to the manufacturer's instructions. First-strand cDNA (1 µl) was used as the template for the polymerase chain reaction (PCR).

Total RNA was isolated from *H. axyridis* adults after cold induction, and 1 μg total RNA was used for subsequent synthesis of first-strand cDNA using the above-described method. The expression levels of select genes from *H. axyridis*, including five soluble trehalases, two membrane-bound trehalases, TPS, GP, and GS were estimated by quantitative real-time PCR (qRT–PCR) using a Bio-Rad CFX96™ system and SsoFast™ EvaGreen^®^ Supermix (Bio-Rad). Next, qRT–PCR was performed in a 20 μl total reaction volume containing 1 µl cDNA sample, 1 µl (10 µmol/µl) of each primer, 7 µl RNase-free and DNase-free water, and 10 µl SsoFastTM EvaGreen^®^ Supermix. Primers were replaced with H_2_O as a negative control and *Harp49* (*H. axyridis* ribosomal protein 49 gene, GenBank Accession No. AB552923) was used as an endogenous control or other reference genes (Olson et al., 2014). The primers were as follows: *Harp49-qF* (5′-GCG ATC GCT ATG GAA AAC TC-3′) and *Harp49-qR* (5′-TAC GAT TTT GCA TCA ACA GT-3′) (Osanai-Futahashi et al., 2012; Shi et al., 2016). Primers for the trehalase genes, TPS, GP, and GS of *H. axyridis* were designed as a part of this study to target unique regions. The primer sequences and annealing temperature of each primer pair are shown in Table 1. The efficiency of target amplification is identical to the efficiency of reference amplification at each annealing temperature. The cycling parameters were as follows: 95°C for 3 min for the initial denaturation, followed by 40 cycles at 95°C for 10 s and 56–62.5°C for 30 s. A melting curve analysis (65°C–95°C) was performed according to the manufacturer's instructions to ensure that only a single product was amplified (SsoFastTM EvaGreen^®^ Supermix; Bio-Rad). Data were analyzed using the relative quantification method (ΔΔC_t_) (Livak and Schmittgen, 2001).

**Table 1.**
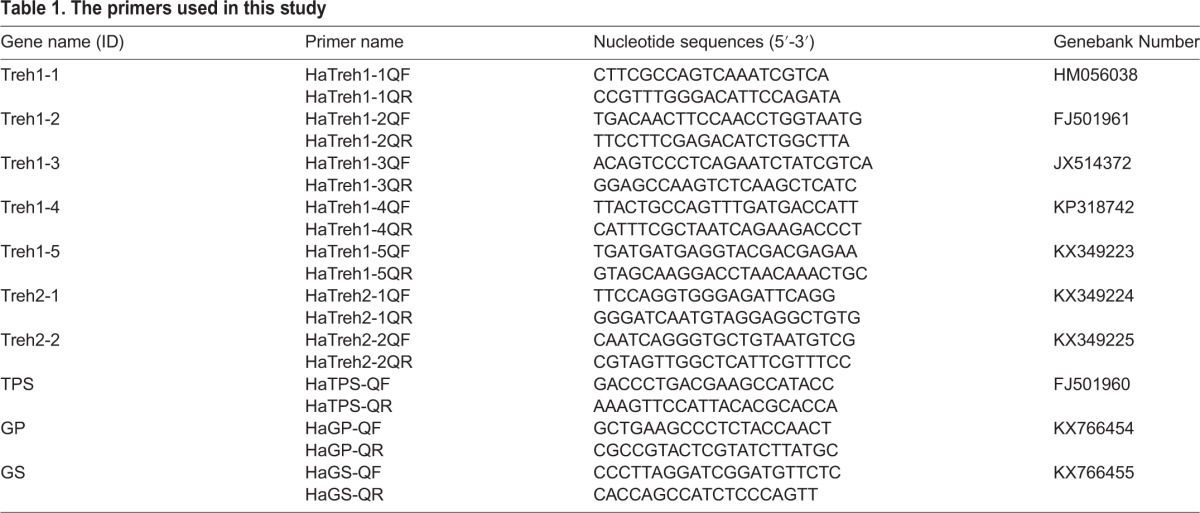
**The primers used in this study**

### Statistical analyses

All the data were presented as the relative mRNA expression (mean±s.d.). Data were evaluated for normality and homogeneity of variance. Trehalose content, glycogen content, the two trehalase activities, and relative mRNA expressions were analyzed using a one-way ANOVA with the Statistica software package version 7.0 (StatSoft Inc., Tulsa, USA). Multiple comparisons of means were conducted using Tukey's test. Differences between means were deemed to be significant when *P*≤0.05.
